# Training needs of German healthcare professionals regarding sexual health and sex workers: results of a nationwide, cross-sectional survey

**DOI:** 10.1186/s12909-024-06551-3

**Published:** 2024-12-30

**Authors:** Sabrina Reinehr, Nina R. Neuendorff, Raquel van der Veen, Benedikt P. Langenbach, Andreas Thieme

**Affiliations:** 1 Experimental Eye Research Institute, University Eye Hospital, Ruhr-University Bochum, Bochum, Germany; 2 Department of Haematology and Stem Cell Transplantation, Faculty of Medicine, University Hospital Essen, University of Duisburg-Essen, Essen, Germany; 3 Department of Geriatrics, Marien Hospital Herne, University Hospital, Ruhr-University Bochum, Herne, Germany; 4 Center for Translational Neuro- and Behavioral Sciences (C-TNBS), Faculty of Medicine, University Hospital Essen, University of Duisburg-Essen, Essen, Germany; 5 Department of Neurology, Faculty of Medicine, University Hospital Essen, University of Duisburg-Essen, Essen, Germany; 6 Department of Psychiatry and Psychotherapy, Faculty of Medicine, LVR-University Hospital Essen, University of Duisburg-Essen, Essen, Germany

**Keywords:** Medical students, Physicians, Psychotherapists, Medical training, Medical education, Personalized medicine, Attitudes of healthcare personnel, Germany

## Abstract

**Background:**

Sexual health is an important aspect of human well-being. In terms of sexual health and healthcare, sex workers might need more specialized care than others, given their higher risk for both discrimination and various sexually transmitted diseases. However, little is known about the quality of healthcare professionals’ training regarding sexual health and healthcare of sex workers in Germany.

**Methods:**

In an online survey, 130 physicians, 63 psychotherapists, and 154 medical students reported their perceived quality of training regarding sexual health problems in all their patients and regarding sex workers’ health issues specifically.

**Results:**

A substantial share of respondents reported to have experienced inadequate training regarding both sexual health problems in general and specific issues concerning sex workers. However, most respondents reported feeling rather comfortable when dealing with these topics. There was a positive correlation between feeling comfortable when treating sexual health problems/ sex workers and the perceived training on these topics.

**Conclusions:**

The results of this study indicate that sexual health issues and sex workers’ specific healthcare requirements are not sufficiently addressed in the curricula of German healthcare professionals. Future adaptations of these curricula might be necessary.

**Supplementary Information:**

The online version contains supplementary material available at 10.1186/s12909-024-06551-3.

## Background

Sexual health and well-being play an important role for overall physical and psychological integrity, health equity, and public health actions [1]. In healthcare, these issues are often neglected, which is surprising given the impact of sexual health on many aspects of health and well-being [2–4]. Healthcare providers’ (HCPs) underutilization of and discomfort regarding sexual history taking was demonstrated to correlate with less training in patient communication [2], having a different gender than the patient [5], fear of intrusion and inadequacy, and cultural differences between HCPs and patients [6]. In addition, with growing impact of personalized medicine [7, 8], knowledge about specific health risks and preventive measures for societal minorities gain importance in healthcare. Sex workers belong to these minorities and have, for example, a higher risk than the general population for infectious diseases, such as human immunodeficiency virus (HIV), human papilloma virus (HPV), viral hepatitis [9, 10], HPV-associated cancers (anal and cervical cancer) [11], and psychological disorders (anxiety disorders, depression) [12]. Therefore, this minority most likely would profit from personalized healthcare including preventive measures [13]. However, no explicit prevention program exists for sex workers in Germany. Instead, it is well-known that numerous barriers hinder sex workers’ healthcare utilization. These barriers include negative experiences with HCPs, anxiety associated with physical exams [14, 15], and a general stigmatization by HCPs [16, 17]. To elucidate possible prejudices towards sex workers in HCPs, we recently conducted a cross-sectional online survey of German HCPs and found a similar prevalence of prejudices towards sex workers as in the general German population [18]. In addition, we found that HCPs overestimated the prevalence of various mental and somatic disorders in sex workers. The latter finding was also influenced by the attitudes of HCPs towards sex work and sex workers. In sum, the results of our foregoing study suggested a knowledge gap among German HCPs about the specific health-related risks of sex workers. This knowledge gap most likely contributes to poor healthcare of sex workers. Previously, and somewhat in line with our findings, others have argued that sexual health is not sufficiently addressed in clinical practice [19, 20], even though our results appear to be rather dated. Thus, as secondary aim of our above-mentioned survey, we evaluated the perceived quality of graduate and postgraduate training in addition to desired training needs on sexual health in general and on sex workers’ health issues specifically among medical students, psychologists, and physicians themselves. We hypothesized that the perceived quality of training is associated with feeling comfortable when treating sexual health problems/sex workers.

## Methods

### Study procedures

This nationwide cross-sectional study was conducted over a period of 90 days in 2022. The online survey was generated with SoSci Survey and provided on www.soscisurvey.de [21]. Participation was restricted to German HCPs as well as medical students. For detailed information, please refer to [18]. Invitations to participate were disseminated through different social media platforms (Instagram and Facebook), via newsletters of universities and healthcare-related societies (e.g., “Berufsverband Deutscher Internisten”/Association of German Internists, “Deutsche Gesellschaft für Verhaltenstherapie”/German Society for Behavioral Therapy), and personal contacts. Participation was restricted to German healthcare workers and medical students. All complete data sets of participants which stated to work in one of the professions of interest (HCPs who were involved in direct patient care) were included in the analysis. Incomplete surveys, and surveys of participants who did not meet the required characteristics (e.g., laboratory personnel without contact with patients) were excluded from the analysis. For the present analysis, only medical students, physicians, or psychologists/psychological psychotherapists were included.

All participants took part voluntarily, did not report their name, and the information provided was not specific enough to identify individual participants. Still, the acquired data were only accessible to the designated researchers, as required by the local ethics committee at the Medical Faculty of the University Duisburg-Essen, Germany (ethics vote no.: 22-10678-BO).

### Questionnaire items

As described previously [18], participants were asked to provide basic demographic information. Measurements of participants’ attitudes to both sex work and sex workers were acquired using the “Attitudes towards Prostitutes and Prostitution Scale (APPS)” by Levin and Peled [22]. In addition, six questions about whether participants felt comfortable with and well-trained about sexual health problems of their patients and sex workers’ health issues specifically were included (see Supplement for questions in German): (1) “Do you feel comfortable when treating sexual problems in your patients?” (2) “Do you feel comfortable when treating sex workers?” (3) “Do you feel well-trained by your graduate training to treat problems of sexual health?” (4) “Do you feel well-trained by your graduate training to treat specific health problems of sex workers?” (5) “Do you feel well-trained by your post-graduate training to treat problems of sexual health?” (6) “Do you feel well-trained by your post-graduate training to treat specific health problems of sex workers?”). Responses were recorded using a 7-point Likert-Scale ranging from “fully disagree” to “fully agree” (1 = fully disagree, 2 = disagree, 3 = somewhat disagree, 4 = neither agree nor disagree, 5 = somewhat agree, 6 = agree, 7 = fully agree).

### Data analysis

Statistical analyses were performed using R (version 4.0.0) [23]. In a first step, we computed descriptive statistics (percentages, mean, median, and standard deviation). To analyze whether age influenced the perception of training or level of comfort when dealing with sexual problems or sex workers, we calculated Pearson’s correlation coefficient. Similarly, we calculated Pearson’s correlation coefficient to check whether perceived training and attitudes towards sex workers were connected. To evaluate the influence of the number of sex workers treated on the perceived quality of training, we used Spearman’s correlation (as the number of sex workers treated had been assessed as an ordinal variable). To analyze the potential effect of gender on perceived levels of comfort, we calculated ANOVAs. P-values below 0.05 were considered significant, with * *p* < 0.050, ** *p* < 0.010, and *** *p* < 0.001.

## Results

### Study sample

In total, 508 participants took part in our study and completed the survey [18]. Of those, 347 were included in the current analysis. The excluded participants fell outside the scope of the article, which aimed to focus on physicians, psychologists, and medical students. However, the survey was also completed by, for example, nurses or social workers. All participants gave their informed consent prior to participation. For demographic details of our sample, see Table 1.

**Table 1:** Participants’ characteristics

Variable	Absolute Number (%), *N* = 347
Medical students	*N* = 154 (44.4%)
Median age (± SD, range)	23.9 (± 3.34, 18–35)
Gender (female/male/other)	116/36/2
Pre-clinical part of their studies (first 4 semesters)	*N* = 41 (26.6%)
Clinical part of their studies (last 8 semesters)	*N* = 109 (70.8%)
No information	*N* = 4 (2.6%)
Physicians	*N* = 130 (37.5%)
Median age (± SD, range)	40.4 (± 11.5, 23–75)
Gender (female/male/other)	84/45/1
Speciality: general practitioner/internal Medicine	47
Speciality: Obstetrics/gynaecology	16
Speciality: Ophthalmology	10
Speciality: psychiatry/psychosomatic medicine	22
Speciality: others	35
Psychologists/psychological psychotherapists	*N* = 63 (18.1%)
Median age (± SD, range)	38.1 (± 11.2, 25–85)
Gender (female/male/other)	49/11/3

### Perceived treatment of sexual health problems and sex workers

**Fig. 1:**
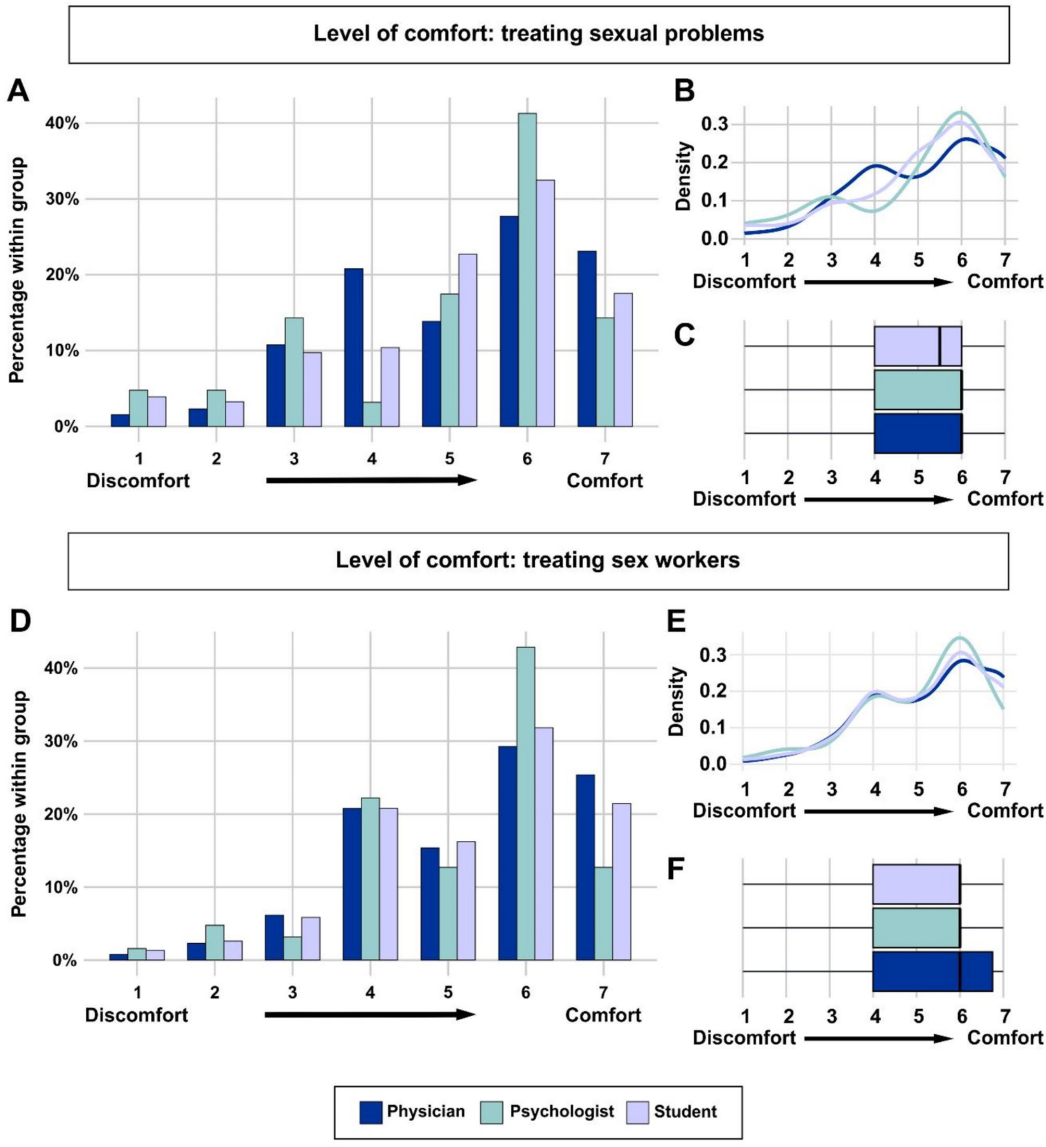
Level of comfort when treating sexual health issues and sex workers. **A-C**: Most participants felt rather comfortable when treating sexual health problems. However, some of participants reported some level of discomfort in this context. **D-F**: Similarly, most participants felt comfortable treating sex workers, while a minority reported at least some level of discomfort in this matter

Most participants felt rather comfortable when treating sexual health problems (~ 70% stated to feel “somehow comfortable” to “strongly comfortable”). However, ~ 17% of participants reported some level of discomfort in this context (Fig. 1A-C).

Similarly, most participants felt comfortable treating sex workers (69.5% stated to feel “somehow comfortable” to “strongly comfortable”), while 9.5% reported at least some level of discomfort in this matter (Fig. 1D-F).

### Perceived training regarding sexual health problems and sex workers

#### Graduate training

**Fig. 2:**
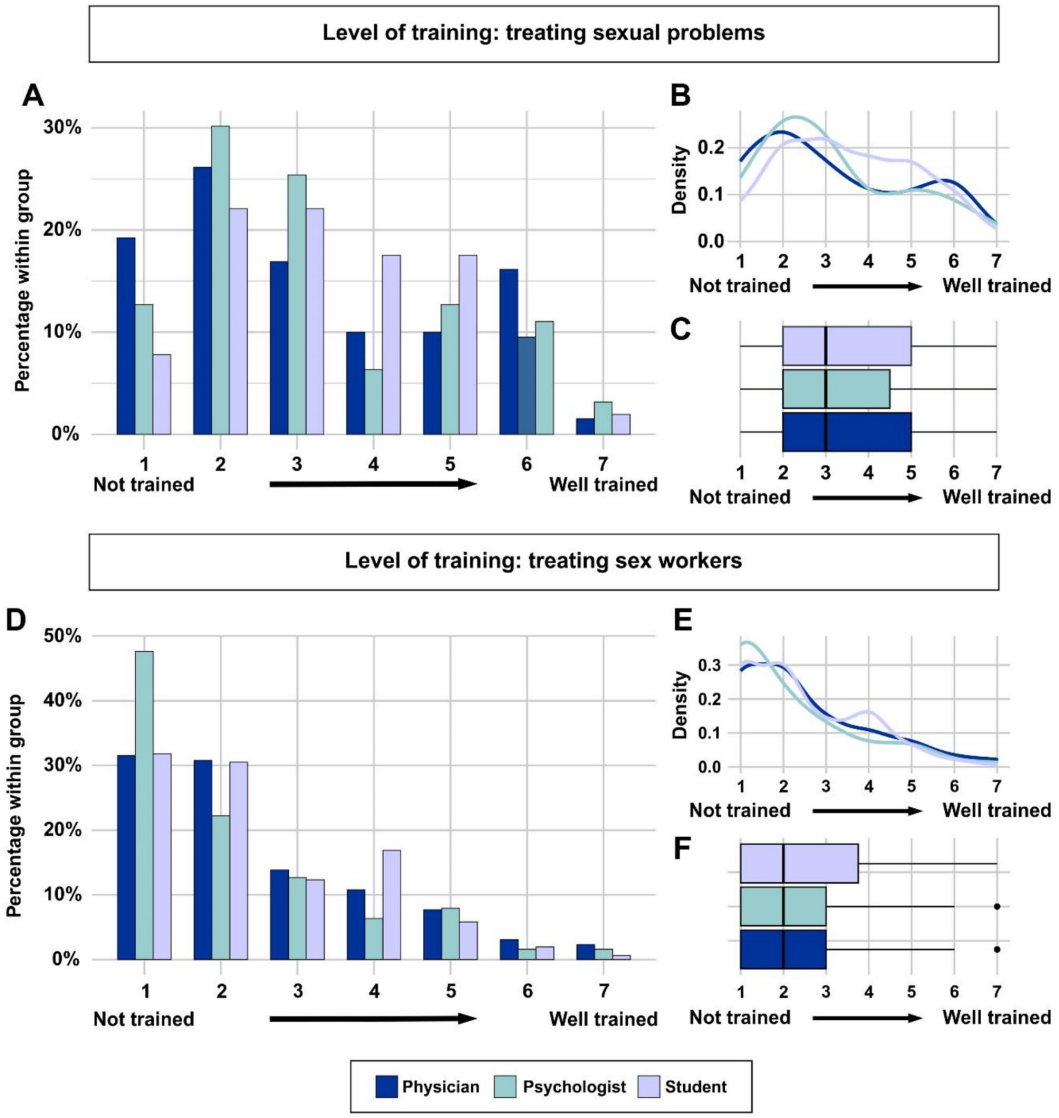
Perceived level of training in treating sexual health issues and sex workers. **A-C**: Of all respondents, almost 60% reported some level of feeling inadequately trained, and almost one in five physicians said they did not feel “trained at all” during their university studies to deal with sexual health problems. **D-F**: Even more clearly, about 34% felt “not trained at all” regarding the specific health problems of sex workers and about 77% reported insufficient training to some degree

In contrast to the overall comfort with the treatment of sexual health problems/sex workers, participants showed a less positive perception regarding the quality of their graduate training regarding sexual health problems and sex workers. Of all respondents, almost 60% reported some level of feeling inadequately trained, and almost one in five physicians (^~^19%) said they did not feel “trained at all” during their university studies to deal with sexual health problems (Fig. 2A-C).

Even more apparently, more than one in three respondents (~ 35%) felt “not trained at all” regarding the specific health problems of sex workers and ~ 77% reported insufficient training to some degree (Fig. 2D-F). Of note, the answers were similar in the subgroups of medical students, psychologists, and physicians. Treating more sex workers did not influence participants evaluation of their training, *r* = 0.004, *p* = 0.9446.

#### Postgraduate training

**Fig. 3:**
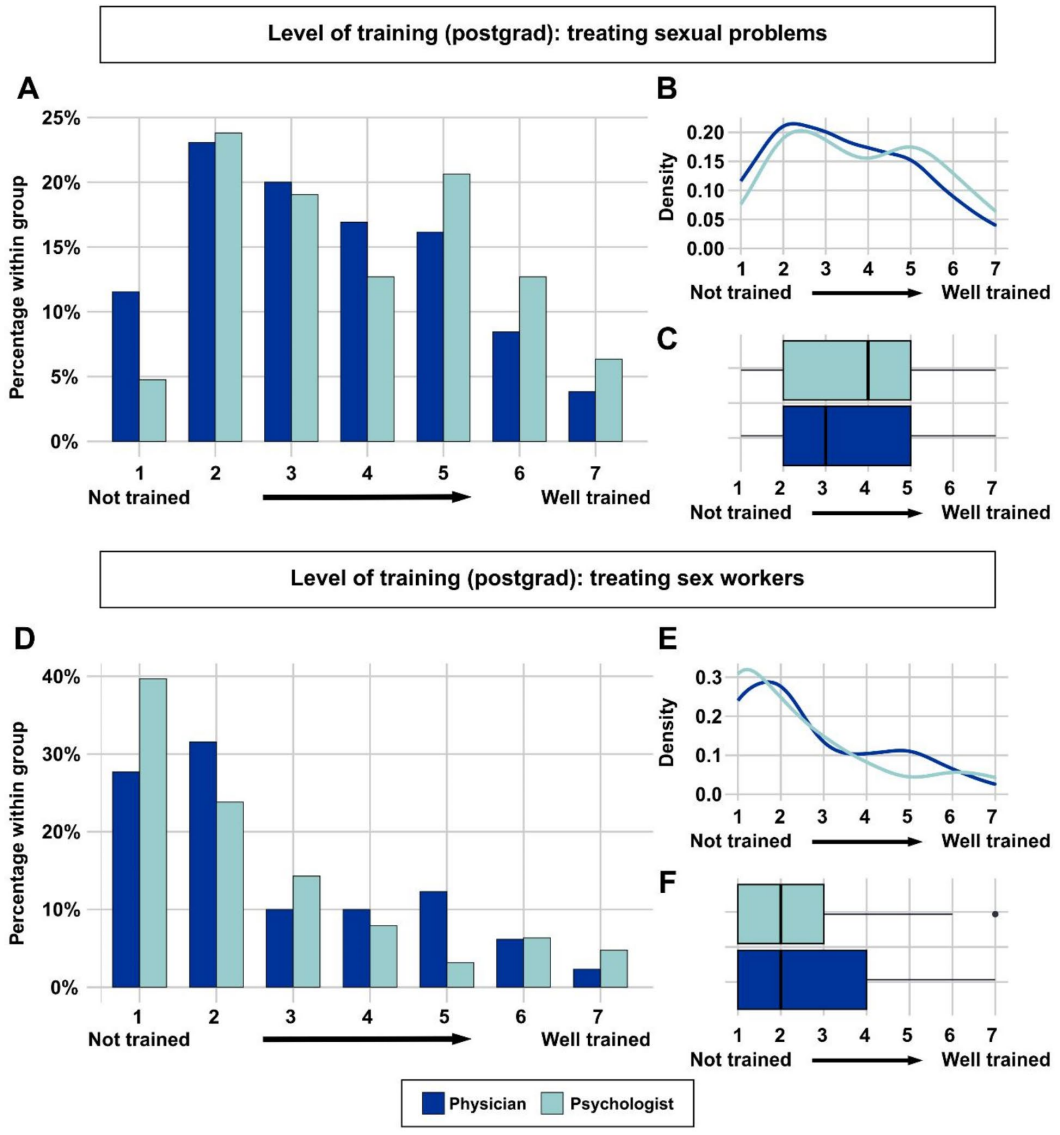
Level of postgrad education in treating sexual health issues and sex workers. **A-F**: A substantial portion of respondents felt that they had received inadequate training regarding sexual health and regarding sex workers’ health issues

We also asked participants about their postgraduate training (i.e., residency for physicians; psychotherapeutic training after receiving M.Sc. for psychologists). Answers were mixed, but a substantial portion of respondents (~ 38%) felt that they had received inadequate training regarding sexual health and ~ 52% regarding sex workers (Fig. 3A-F). Of note, psychologists apparently felt better trained during their postgraduate training regarding sexual health issues than physicians (with ~ 40% versus ~ 28% feeling at least to some extent adequately trained). Again, treating more sex workers did not influence participants evaluation of their postgraduate training, *r* = -0.034, *p* = 0.5272.

### Influence of age on level of comfort and perceived training regarding treating sexual health problems and sex workers

To evaluate whether age was connected to participants’ comfort and their perceived level of training on the matter, we conducted additional correlation analyses: First, we analysed whether age was related to the reported level of feeling (un)comfortable when treating sexual health problems in general or when treating sex workers, respectively. Second, we analysed separately whether the perceived level of education during academic/graduate (i.e. university studies) and postgraduate training regarding sexual health problems and sex workers was influenced by age. There was no relation between age and feelings while treating sexual health problems, *r* = 0.013, *p* = 0.805, or sex workers, *r* = -0.058, *p* = 0.282. Similarly, there was no relation between age and perceived quality of academic training regarding sexual health, *r* = 0.077, *p* = 0.151, or sex workers, *r* = 0.116, *p* = 0.108. Finally, there was no relation between age and academic training regarding sexual health, *r* = 0.012, *p* = 0.825. While there was a statistically significant connection between age and perceived level of postgraduate training regarding sex workers, *r* = 0.14, *p* = 0.047, this finding did not remain significant after conducting the Bonferroni-correction for multiple tests. Having a different gender from one’s patient has been shown to correlate with a practitioner’s level of feeling (un-)comfortable when discussing sexual health. Thus, we wanted to check whether practitioners’ gender also affected the reported level of feeling comfortable in our study, using ANOVAs with gender as independent variable and levels of feeling comfortable as dependent variable. However, no statistically significant gender differences emerged, neither for physicians, F(2,127) = 0.757, *p* = 0.471, nor for psychotherapists, F(2,60) = 2.150, *p* = 0.125, nor for medical students, F(2,151) = 0.297, *p* = 0.744.

### Correlation between level of comfort and perceived training regarding treating sexual health problems and sex workers

We also analysed whether the level of comfort when treating sexual health problems or sex workers correlated with the perceived training on these topics. Indeed, a small statistically significant correlation was detected between the level of comfort when treating sexual health problems and the perceived training, both for graduate training, *r* = 0.24, *p* = 0.000, and for post-graduate training *r* = 0.30, *p* = 0.000.

Similarly, there was a small, but statistically significant correlation between the level of comfort when treating sex workers and the perceived training, again both for graduate, *r* = 0.19, *p* = 0.000, and for post-graduate training, *r* = 0.23, *p* = 0.001. (For analysis of post-graduate training, students were not included as they – for obvious reasons – did not receive post-graduate training).

### Correlation between perceived training and attitudes towards sex workers

Finally, we analyzed whether the perceived level of training was related to participants’ attitudes towards sex workers. Interestingly, we found no connection between participants’ self-reported quality of training regarding sex workers and their estimation of sex workers as victims vs. self-choosing agents, neither for graduate, *r* = -0.07, *p* = 0.185, nor for post-graduate training, *r* = -0.12, *p* = 0.085.

Similarly, there was no connection between the perceived level of training and participants’ ratings of sex workers as (morally) deviant vs. normative, neither for graduate, *r* = 0.046, *p* = 0.39, nor for post-graduate training *r* = -0.081, *p* = 0.26. (Again, for analysis of post-graduate training, students were not included).

## Discussion

This cross-sectional online survey examined how comfortable German HPCs feel when treating sexual health problems and sex workers. Moreover, their perceived training on these topics was assessed.

The main findings were (1) that most participants felt rather comfortable when treating sexual health problems or sex workers, (2) that most participants reported not being well-trained on these topics, (3) that there was a significant correlation between feeling comfortable when treating sexual health problems/sex workers and the perceived training regarding these topics. Moreover, our study found (4) no correlation between the perceived training and attitudes towards sex workers.

Several studies have shown that the lack of training is one reason for HCPs’ discomfort in regard to diagnosis and treatment of sexual health issues [24–26]. For example, one study conducted in ~ 60 medical students in the United States found that participation in sex education improved self-efficacy in discussing nine sexual health topics and in performing nine advanced interviewing skills relevant to sexual healthcare [25]. Another study, which included 190 medical students, 75 residents and 11 fellows in the United States yielded similar results: only residents in the field of urology and obstetrics/gynaecology felt confident in their ability to assist patients with a sexual health issue. All other trainees lacked confidence in attending to sexual health concerns, regardless of their training level [26]. Our finding that participants felt rather comfortable when treating sexual health issues in general or sex workers specifically is a bit surprising because most participants reported that they felt rather poorly trained on these topics. According to the literature [24–26], one would expect that our cohort would feel rather uncomfortable when treating patients with sexual health issues. One explanation to that could be that most of our study participants stated to treat very few or no sex workers [18], implicating that they maybe did not know the (real) profession of their respective patients. In Germany, sex work is legal and ~ 30,000 sex workers were officially registered in 2023 [27]. Presumably, this number underestimates the real prevalence due to forced prostitution and/or illegal residence of sex workers. Thus, we expect that our participants regularly were in contact with sex workers but may not have been aware of it . Other factors (which have not been captured in this study) might also have contributed to the perception of feeling comfort when treating sexual health issues and sex workers [28]. It is possible that personality traits such as open-mindedness and tolerance influence the perception when treating stigmatized societal groups such as sex workers. Also, work experience might play a role [4, 24]. Healthcare personnel is repeatedly confronted with challenging situations throughout their professional life. This likely makes them resilient to handle difficult situations including demanding conversations.

However, like initially mentioned, knowledge deficits likely exist when it comes to treatment of sexual health issues and sex workers. In our foregoing study (with the same participants), we have shown that German HCPs have objectifiable knowledge deficits about the specific health risks of sex workers [18]. This is in line with a recent German survey among ~ 260 medical students which assessed basic knowledge about sexual health and found major deficits on important aspects, such as psychosexual development and relative safety of different contraceptives [29]. In the present analysis, a high percentage of German HPCs reported to be only poorly trained regarding the treatment of sexual health issues or sex workers, but we found a positive correlation between feeling comfortable when treating sexual health problems or sex workers and the perceived level of training. In other words, HCPs which felt to be better trained on these topics were more comfortable when treating the respective patients. Also, it might be possible that participants received additional (voluntary) training regarding sexual health outside their formal training after which we inquired, leading to them feeling more at ease when discussing sexual health. This observation is in line with similar results from the study of Mkonyi and colleagues which was conducted in ~ 60 healthcare professionals and ~ 60 students of healthcare professions in Tanzania: The confidence of medical history-taking regarding sexual health problems increased with professional experience [24].

The current results might be important for evaluating medical curricula. Indeed, specialized medical societies have argued for more room for sexual health in medical education [19, 30]. Our results support their claim since HCP themselves often regard their training as insufficient.

In our study, interestingly no correlation was found between participants’ perceived training regarding sex workers and their estimation of sex workers as victims vs. self-choosing agents. Similarly, there was no correlation between the perceived training and participants’ ratings of sex workers as (morally) deviant vs. normative. This contrasts with results from a study from Hong Kong conducted in ~ 320 nursing students which found a relationship between nursing students’ knowledge of and attitudes against sex workers. In that study, final-year students (who were assumed to have received more training on sex workers’ specific health issues) had more positive attitudes towards sex workers than first-year students [31]. The contrasting results of our study may have several reasons. Firstly, it is known that information alone often is not sufficient to change stereotypes and, secondly, it is known that stereotypes depend on societal norms [32]. The latter most likely differs between Hong Kong and Germany which makes a direct comparison difficult in this matter.

Although this study delivers evidence that German HCPs feel only poorly trained concerning the treatment of sexual health issues in general and sex workers specifically, the study has some limitations. First, there were challenges with outreach and recruitment of study participants. Though recruitment e-mails were sent to related societies (German Society for Behavioral Therapy, Association of German Internists) and invitations were disseminated through social media platforms and newsletters of universities, unidirectional nature of this study limited the ability to discern how many healthcare workers received the invitation to participate. Therefore, we do not have information about non-responders in this study. In general, potential reasons for non-response include lack of interest, missing access to the questionnaire, perceived lack of personal relevance of the topic or an aversion to the subject of sex work. Secondly, despite the sample size was larger than in comparable studies, the current sample size might be too small to generalize the findings to all HCPs in Germany. For example, in our study, 130 physicians participated, compared to ~ 428.000 active physicians in Germany in 2023, hence, our sample represented only ~ 0.03% of all physicians [33]. The according ratios for medical students (~ 0.1%) [34] and psychotherapists (~ 0.2%) [35] were similar. Also, our sample was skewed towards younger participants, females as well as some subdisciplines (e.g. general practitioners/internal medicine, obstetrics/gynaecology, psychiatry/psychosomatic medicine, and medical students were overrepresented; see Table 1). To gain at least a basic impression of the generalizability of our data, we compared age and gender distribution in our sample to that of the parent population. With a mean age of 40 years, the physicians in our study were close to the average age of a physician working in a hospital (41 years), although those working in ambulatory settings are older (54 years). Similarly, with a mean age of 38 years, the psychotherapists in our sample were close to the average age of employed psychotherapists (41 years for both those working in clinics and those employed in psychotherapeutic practices), yet somewhat younger than those owning their own practice (55 years) [36]. To the best of our knowledge, there is no public statistic on the average age of medical students. However, with a mean age at graduation of 26 years in 2023, the mean age of 24 years in our sample might be close to the actual population value [37]. Regarding gender distribution, our sample had 50%, 78%, and 75% women for physicians, psychotherapists, and medical students, respectively. In the respective parent populations, these values are 46% [38], 77% [39], and 65% [40], making our sample overall comparable, perhaps except for a slightly more “female” student sample. Thus, both physicians and psychotherapists in our sample differ somewhat from the population at large. Getting a representative sample of German psychotherapists, physicians, and medical students, however, would be extremely difficult (if possible, at all). Moreover, our results might not be free of a self-selection bias. This bias is known to play a role in surveys where the participants themselves chose to participate [41]. It is possible that individuals with a special interest in the topic participated in this study more frequently. Also, we cannot fully rule out that single duplicate responses were within in the final data set. Due to data protection regulations, comprehensive tracking of duplicate study participations was not possible. However, in the optional lottery following study participation duplicate responses were only spotted twice within the sample of 115 individuals who participated in the lottery, yielding a possible rate of duplicate responses of < 2%. We believe that this is representative for the whole sample of all 508 participants and that duplicate responses (if they occurred at all) did not influence the results significantly.

The current findings complement and extend the results of our foregoing study which found evidence for knowledge gaps in German HCPs regarding the specific health risks of sex workers. Taken together, improvements in graduate and postgraduate training of German HCPs about the specific health risks of sex workers and sexual health problems in general seem necessary. Indeed, the NKLM (Nationaler Kompetenzbasierter Lernzielkatalog Medizin), which is a national competency-based catalogue of objectives for medical education, picks up some of the necessary learning goals that could help to improve care of sex-related health issues and of sex workers. The NKLM was developed in response to the 2008 recommendations of the German Council of Science, aiming to improve the quality of medical education in Germany. While legal frameworks provided a basic structure for medical education, they were insufficient to adequately define the specific competencies required of medical graduates. The NKLM 1.0 was released in 2015, and its further development led to the creation of the NKLM 2.0 in 2021. Although in the NKLM 2.0, no competencies specifically address the need of sex workers, some competencies deal with sexual and mental health issues, maltreatment and abuse, drug addition, medical history taking and socio-ethnic features [42]. All these competencies appear helpful to improve the healthcare of sex workers and sex-related health issues. However, specific learning goals for care of sex workers (or other societal marginal groups) are missing.

## Conclusions

Sexual health is an important aspect of human well-being. For sex workers, adequate medical care might often be inaccessible, and stigmatization seems common. Thus, for both these topics, medical professionals should receive adequate training. While the German national competency-based catalogue of objectives for medical education (NKLM 2.0) already contains some helpful learning goals to manage sex-related health issues, specific learning goals for the treatment of sex workers (or other marginal societal groups) are missing. We recommend that specific learning goals for sex workers shouldbe implemented in the next version of the NKLM. Moreover, sexual health and healthcare of sex workers should be more prominently addressed in post-graduate training within the medical sector.

## Electronic supplementary material

Below is the link to the electronic supplementary material.

Supplementary Material 1

## Data Availability

The datasets used and/or analyses performed in the current study are available from the corresponding author upon reasonable request.

## Abbreviations

HCPs: Health care providers
HIV: Human immunodeficiency virus
HPV: Human papilloma virus
APPS: Attitudes towards prostitutes and prostitution scale
