# Supplementary material for: Training needs of German healthcare professionals regarding sexual health and sex workers: results of a nationwide, cross-sectional survey
Source: BMC Med Educ. 2024 Dec 30;24:1557. doi: 10.1186/s12909-024-06551-3 (PMC11686851; doi:10.1186/s12909-024-06551-3)
Supplement: Supplementary file 1 — Supplementary Material 1 [file 12909_2024_6551_MOESM1_ESM.docx]

# **Supplementary material to:**

# **Training needs of German healthcare professionals regarding sexual health and sex workers: Results of a nationwide, cross-sectional survey**

Sabrina Reinehr, Nina R. Neuendorff, Raquel van der Veen, Benedikt P. Langenbach, Andreas Thieme

**Applied questionnaire in German (English translation in parenthesis):**

1. Fühlen Sie sich wohl im Umgang mit sexuellen Problemen bei Patient:innen?

(Do you feel comfortable when treating sexual problems in your patients?)

1. Fühlen Sie sich wohl im Umgang mit Sexarbeiter:innen?

(Do you feel comfortable when treating sex workers?)

1. Fühlen Sie sich in Studium/ Ausbildung adäquat ausgebildet, um mit Problemen der sexuellen Gesundheit umzugehen?

(Do you feel well-trained by your graduate training to treat problems of sexual health?)

1. Fühlen Sie sich in Studium/ Ausbildung adäquat ausgebildet, um mit spezifischen gesundheitlichen Problemen von Sexarbeiterinnen umzugehen?

(Do you feel well-trained by your graduate training to treat specific health problems of sex workers?)

1. Fühlen Sie sich während der Weiterbildung im Fachbereich adäquat ausgebildet, um mit Problemen der sexuellen Gesundheit umzugehen?

(Do you feel well-trained by your post-graduate training to treat problems of sexual health?)

1. Fühlen Sie sich während der Weiterbildung im Fachbereich adäquat ausgebildet, um mit spezifischen gesundheitlichen Problemen von Sexarbeiterinnen umzugehen?

(Do you feel well-trained by your post-graduate training to treat specific health problems of sex workers?)
